# CD1 molecules: Beyond antigen presentation

**DOI:** 10.1016/j.molimm.2024.03.011

**Published:** 2024-06

**Authors:** Lauren Evans, Patricia Barral

**Affiliations:** a The Peter Gorer Department of Immunobiology. King’s College London, London, UK; b The Francis Crick Institute, London, UK

**Keywords:** CD1, Lipid metabolism, CD1d crosslinking

## Abstract

CD1 molecules are well known for their role in binding and presenting lipid antigens to mediate the activation of CD1-restricted T cells. However, much less appreciated is the fact that CD1 molecules can have additional “unconventional” roles which impact the activation and functions of CD1-expressing cells, ultimately controlling tissue homeostasis as well as the progression of inflammatory and infectious diseases. Some of these roles are mediated by so-called reverse signalling, by which crosslinking of CD1 molecules at the cell surface initiates intracellular signalling. On the other hand, CD1 molecules can also control metabolic and inflammatory pathways in CD1-expressing cells through cell-intrinsic mechanisms independent of CD1 ligation. Here, we review the evidence for “unconventional” functions of CD1 molecules and the outcomes of such roles for health and disease.

## 1 Introduction

The CD1 family consists of 5 antigen presenting molecules structurally and functionally related to Major histocompatibility complex class I (MHC-I) and II (MHC-II) molecules (Ogg et al., 2019). The family is subdivided into group 1 (CD1a, CD1b, CD1c and CD1e) and group 2 (CD1d) based on sequence homology. Unlike MHC-dependent presentation of peptides to conventional T cells, CD1 molecules bind and present lipid antigens to CD1-restricted T cells (also called NKT cells) (Mori et al., 2016). The lipid presentation system is fine-tuned such that each of the CD1 family members have unique properties in terms of expression (at a cellular, tissue and species level), lipid binding and intracellular recirculation (Chancellor et al., 2018, Moody and Cotton, 2017, Vartabedian et al., 2016). While CD1 molecules are monomorphic, they have the capacity to bind and present a vast array of endogenous or microbial lipids triggering the activation of CD1-restricted T cells (Huang et al., 2023). As such, based on CD1 restriction and T cell receptor (TCR) specificity, NKT cells can be classified as type I (invariant, iNKT) or type II. iNKT cells recognise the lipid α-galactosylceramide (α-GalCer) via their semi-invariant TCR and can be identified using α-GalCer-loaded CD1d tetramers in both human and mouse. In comparison, type II NKT cells possess a diverse TCR repertoire and recognise a broader range of lipids presented by CD1a-d (Godfrey et al., 2015). Much progress has been made in recent years toward understanding the activation and functions of CD1-restricted T cells and to harness their therapeutic potential in many contexts including infection, cancer or autoimmune disease (Exley et al., 2021).

**Fig. 1:**
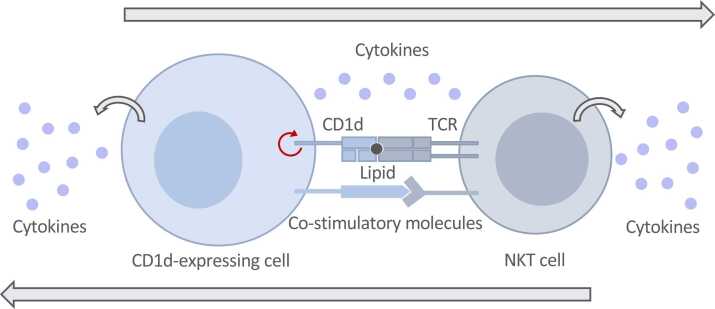
**The functions of CD1d.** The CD1d-NKT axis is not unidirectional and leads to the activation of NKT cells and CD1d-expressing cells. CD1d-dependent presentation of lipids to NKT cells results in NKT cell activation, but also in the direct provision of “help” to CD1d-expressing cells. Antigen presenting cell (APC)-NKT cell interactions can be supported by the engagement of costimulatory molecules and cytokines. In addition, reverse CD1d signalling also likely contributes to APC activation. TCR, T cell receptor.

**Fig. 2:**
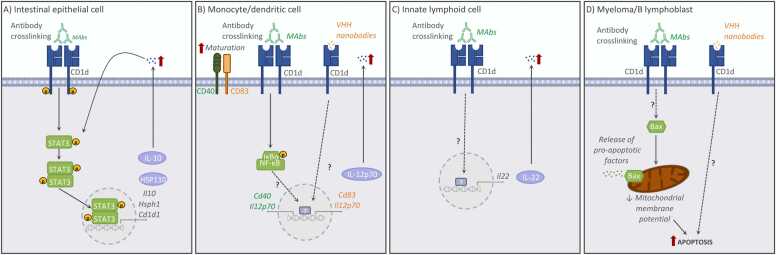
Summary of CD1d functions mediated by reverse signalling following antibody crosslinking at the cell surface of various CD1d-expressing cell types. The main signalling pathways in **(A)** murine intestinal epithelial cells (Olszak et al., 2014), **(B)** human monocytes and dendritic cells (Yue et al., 2005, Lameris et al., 2016), **(C)** murine group 3 innate lymphoid cells (Saez de Guinoa et al., 2017) or **(D)** human myeloma and B lymphoblastoid cell lines (Spanoudakis et al., 2009, Lameris et al., 2016) are shown.

**Fig. 3:**
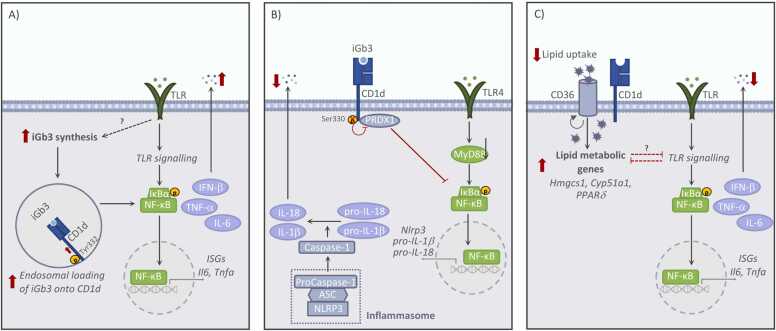
Summary of the cell-intrinsic functions of CD1d in murine macrophages (independent of receptor ligation, antibody crosslinking or NKT cells). The main CD1d-dependent signalling pathways are shown as reported by **(A)**Liu et al., (2019), **(B)**Cui et al., (2020) and **(C)**Brailey et al., (2022). iGb3, Glycolipid isoglobotrihexosylceramide; TLR, Toll-like receptor.

While the biology and structure of CD1 molecules have been extensively investigated, it has recently become apparent that CD1 molecules can control immune responses through various alternative mechanisms. In addition to the well established functions of CD1d molecules in lipid presentation to NKT cells, recent literature supports “unconventional” roles for CD1d (Fig. 1), which can regulate the activation and function of a diverse range of CD1d-expressing cells via two main mechanisms. Firstly, an underappreciated function of CD1d molecules is their ability to signal outside-in (also called reverse signalling) such that ligation of CD1d molecules on the cell surface by the NKT-TCR or crosslinking antibodies can lead to signal transduction and intracellular signalling in antigen presenting cells (APCs, Fig. 2). Secondly, CD1d has also been shown to control inflammatory responses in immune cells by cell-intrinsic regulation of lipid metabolic programs in APCs (Fig. 3).

In this manuscript, we review the evidence for “unconventional” roles of CD1 molecules and the functional outcomes of such roles in tissue homeostasis and disease. While the vast majority of available data pertain to CD1d molecules, it may also carry implications for group 1 CD1 molecules which are also discussed in this paper.

## 2 CD1d molecules: expression, trafficking and reverse signalling

CD1d is the sole member of the group 2 CD1 molecules and is the only CD1 isoform expressed in mice, whereby protein expression is encoded by two homologous genes, *Cd1d1* and *Cd1d2*. In B6 mice the *Cd1d2* gene contains a frame shift mutation, abolishing *CD1d2* protein expression in this strain, while in BALB/c mice, the reading frame for *Cd1d2* allows its transcription, and its expression at the cell surface. While *Cd1d1* is the predominant isoform expressed in peripheral tissues, both molecules have been suggested to regulate the development and function of iNKT cells in the mouse thymus. Here, *Cd1d1* and *Cd1d2* are expressed equally but based on confirmational changes in their lipid-binding clefts, present a different repertoire of antigens to iNKT cells during thymic selection (Sundararaj et al., 2018). Nevertheless, the contribution of the two *Cd1d* isoforms *vs.* intrinsic signals to iNKT cell development and differentiation remain under investigation (Amable et al., 2023).

In contrast to group 1 CD1 molecules (whose expression are limited to professional APCs), CD1d is expressed by a diverse range of cells including hematopoietic (thymocytes, dendritic cells (DCs), B cells, monocytes, innate lymphoid cells (ILCs)) and non-hematopoietic cells (intestinal epithelial cells (IECs), keratinocytes, hepatocytes). Murine and human CD1d molecules share more than 60% homology at the protein level, and like other CD1 isoforms, consist of three extracellular domains, a transmembrane domain and intracellular cytoplasmic tail, and form a heterodimer with β2-microglobulin (Koch et al., 2005). CD1d molecules are synthesized and assembled in the endoplasmic reticulum (ER), where they are loaded with ER-derived lipids in a process involving microsomal triglyceride transfer protein (Brozovic et al., 2004, Dougan et al., 2005). Then, CD1d molecules are transported to the plasma membrane through the Golgi. Upon reaching the plasma membrane, CD1d is internalized and traffics through the endocytic system, allowing it to sample a diverse range of lipid antigens in intracellular compartments. Both human and murine CD1d display a tyrosine-based sorting motif (YXXZ; Y is a tyrosine, X is any amino acid and Z is a bulky hydrophobic amino acid) within the intracellular tail that regulates the trafficking of CD1d through intracellular compartments. As such, intracellular tail deleted CD1d mutants display impaired internalization and trafficking (Chiu et al., 2002, Lawton et al., 2005). In addition to the tyrosine-based sorting motif, human but not murine CD1d also has a threonine in its intracellular tail which has been suggested to control surface expression and intracellular trafficking (Liu et al., 2010). Also, ubiquitination of a lysine residue (Lys^326^) located in the cytoplasmic tail of CD1d has been linked to its endocytosis and subsequent immune evasion in a model of Herpesvirus infection (Sanchez et al., 2005). On the other hand, CD1d can also be directed to late endosomes in murine B cells and DCs through interactions with invariant chain (Ii) in a process independent of the intracellular tail (Jayawardena-Wolf et al., 2001). Within the endocytic pathway, CD1d undergoes lipid exchange such that natural endogenous lipids can be replaced by antigenic endogenous or exogenous lipids. This process of lipid exchange can be facilitated by proteins such as saposins or Niemann-Pick type C2 (Schrantz et al., 2007, Yuan et al., 2007). Once CD1d has undergone lipid transfer, it is recycled back to the cell surface where it can function as an antigen presenting molecule.

CD1d-dependent presentation of lipids to NKT cells results not only in NKT cell activation but also in the direct provision of “help” to the CD1d-expressing cell (Fig. 1). These APC-NKT cell interactions will therefore regulate the activation and function of a variety of immune and non-immune CD1d-expressing cells including B cells, DCs or epithelial cells (Barral et al., 2008, Chang et al., 2011, Fujii et al., 2003, Leadbetter et al., 2008, Olszak et al., 2014, Semmling et al., 2010). The cognate NKT cell help can be mediated by the engagement of co-stimulatory molecules and/or cytokines, but it is likely that CD1d reverse signalling may also contribute to APC activation. Indeed, part of the evidence for reverse CD1d-signalling is supported by the notion that tyrosine and serine residues located in the intracellular tail of CD1d can be phosphorylated and consequently initiate downstream signal transduction and intracellular signalling in APCs (Cui et al., 2020, Olszak et al., 2014). This cognate NKT cell help can, at least in part, be mimicked by direct ligation of CD1d using CD1d-specific monoclonal antibodies. In fact, as discussed in the following sections, direct CD1d-crosslinking is sufficient to control the activation and function of a variety of cell types (Fig. 2). Caution should be employed however when using αCD1d antibodies (particularly *in vivo*), as while some clones may block lipid presentation and NKT cell activation, other clones may induce potent systemic responses mediated by CD1d ligation on APCs (Table 1). For instance, a battery of anti-human CD1d-specific camelid-derived variable domain of heavy-chain-only (VHH) antibodies yielded a diverse array of effects on various CD1d-expressing cells. Two clones induced maturation of DCs, one clone induced apoptosis in B lymphoblasts and another clone blocked recognition of lipid-loaded CD1d by NKT cells (Lameris et al., 2016). Nonetheless, αCD1d antibodies, either by blocking NKT cell functions or by activating CD1d-expressing cells, can provide potential therapeutic options to manipulate immune responses in a variety of diseases as discussed below.

**Table 1:** CD1d antibodies and their downstream effects following ligation in different cell types.

**Reactivity**	**Clone(s)**	**Cell type**	**Effect**	**Reference**
Mouse anti-human CD1d	51.1	IEC	↑ IL-10	Colgan et al., (1999)
Mouse anti-human CD1d	51.1	Epithelial cells (reproductive tract)	↑ IL-12, ↑ IL-15	Kawana et al., (2008)
Mouse anti-human CD1d	51.1	Monocytes	↑ IL-12	Yue et al., (2005)
Mouse anti-human CD1d	51.1 42.1	B lymphoblastoid cell line Myeloma cell line	Apoptosis	Spanoudakis et al., (2009)
Camelid anti-human CD1d (VHH)	VHH2 VHH5	moDC	↑ IL-12, maturation	Lameris et al., (2016)
Camelid anti-human CD1d (VHH)	VHH17	B lymphoblastoid cell line	Apoptosis	Lameris et al., (2016)
Rat anti-mouse CD1d	1B1	IEC	↑ IL-10	Olszak et al., (2014)
Rat anti-mouse CD1d	1B1	ILC3	↑ IL-22	Saez de Guinoa et al., (2017)
Rat anti-mouse CD1d	1B1 3C11	Splenocytes	↑ IL-12, ↑ IFNγ	Yue et al., (2010)
Rat anti-mouse CD1d	19G11	Macrophages	Lipid metabolism, ↑TLR responses	Brailey et al., (2022)
Rat anti-mouse CD1d	3C11 1B1 HB323	*In vivo*	Protection from viral infection	Yue et al., (2010)
Rat anti-mouse CD1d	1B1	*In vivo*	Tumour suppression	Teng et al., (2009)

## 3 CD1d reverse signalling: regulation of epithelial cell function

The first report about CD1d reverse signalling was described in IECs. Expression of CD1d has been identified on IECs from humans, mice and rats. Initial studies suggested that CD1d found on the cell surface of human IECs was non-glycosylated and not associated with β2- microglobulin (Balk et al., 1994). However, later reports identified a β2-microglobulin-associated form of CD1d localized to the apical and lateral membrane domains of polarized epithelia (Somnay-Wadgaonkar et al., 1999). Studies performed with the human epithelial cell line T84, demonstrated that crosslinking of surface CD1d (with antibodies) was sufficient to induce production of IL-10 by these cells (Colgan et al., 1999). This phenotype was dependent on the CD1d cytoplasmic tail, as crosslinking of a chimeric molecule expressing extracellular CD1d and cytoplasmic CD1a did not induce IL-10 secretion (note that the CD1a tail lacks the YXXZ motif). Further evidence for a function for CD1d-intrinsic signalling in epithelial cells has been provided for human vaginal and penile urethral cells. In this context, ligation of surface-expressed CD1d by monoclonal antibody crosslinking induced IL-12 and IL-15, but not IL-10 production (Kawana et al., 2008).

The CD1d-dependent production of IL-10 by IECs and its relevance in immune responses have been more recently demonstrated in the context of intestinal inflammation. In the murine model of oxazolone-induced colitis, CD1d expressed on bone marrow-derived cells elicits pathogenic effects by mediating activation of NKT cells, while CD1d expression by IECs leads to protection mediated by IL-10 production (Olszak et al., 2014). This phenotype is dependent on CD1d reverse signalling: antibody crosslinking of CD1d on IECs (or co-culture with NKT cells) leads to phosphorylation of Signal transducer and activator of transcription 3 (STAT3) and STAT3-dependent expression of IL-10, heat shock protein 110 (HSP110) and CD1d itself (Fig. 2A). Conversely, specific deletion of CD1d in IECs, results in reduced IL-10 production and severe oxazolone-mediated colitis (Olszak et al., 2014). Strikingly, STAT3 activation and IL-10 secretion are again dependent on the CD1d cytoplasmic tail, although the relative contribution of the CD1d-tail deletion to impaired CD1d trafficking *vs.* the loss of retrograde signalling is unclear. Further studies demonstrated that the CD1d-dependent production of IL-10 by IECs in response to oxazole is regulated through the aryl hydrocarbon receptor pathway (Iyer et al., 2018), providing additional mechanistic evidence of the physiological relevance of the CD1d-dependent functions in IECs. Thus, it is clear that CD1d is key to controlling the function and activation of IECs, ultimately regulating intestinal immunity. Remarkably, CD1d expression has been reported to be reduced in the intestine of patients with inflammatory bowel diseases (IBD) (Perera et al., 2007), supporting the idea that reverse signalling through CD1d could be a key regulator of the host response to environmental stimuli and potentially contribute to the control of intestinal inflammation.

## 4 CD1d reverse signalling: immune cell activation and survival

The role of CD1d in the regulation of immune cell activation has been well established. One of the first examples of CD1d having non-classical functions was described using human peripheral blood monocytes and dendritic cells (Fig. 2B). In this case, CD1d ligation (with crosslinking antibodies) was sufficient to induce secretion of IL-12 in a process mediated by nuclear factor kappa-light-chain-enhancer of activated B cells (NFκB) signalling (Yue et al., 2005). Comparable results were observed using CD1d-specific camelid-derived VHHs, which induced maturation and cytokine production by human monocyte-derived dendritic cells (Lameris et al., 2016). Similar activatory functions of CD1d crosslinking have been described for ILCs (Saez de Guinoa et al., 2017) (Fig. 2C). CD1d is expressed in all murine ILC populations, but surface CD1d levels are particularly high in group 3 ILCs (ILC3). Primary murine ILC3 are able to internalize lipid antigens and load them in CD1d, while crosslinking of CD1d (with antibodies) results in ILC3 activation and secretion of IL-22 (Saez de Guinoa et al., 2017). In addition to its activatory function, CD1d crosslinking has been also shown to impact cellular survival (Fig. 2D). CD1d is highly expressed in premalignant and early myeloma, but its expression decreases with disease progression. Engagement of CD1d by anti-CD1d monoclonal antibodies induces cell death of myeloma cell lines associated with overexpression of proapoptotic Bax and mitochondrial membrane potential loss. This effect is dependent on the CD1d cytoplasmatic tail, yet not on the Tyr residue from the sorting motive (Spanoudakis et al., 2009). In keeping with this, an independent study demonstrated that all-trans retinoic acid (ATRA) induces CD1d expression in the human leukaemia cell line HL-60, while ligation with αCD1d antibodies of ATRA-treated cells resulted in increased apoptosis (Ozeki and Shively, 2008). Moreover, CD1d-specific camelid-derived VHHs also induced apoptosis of CD1d-expressing B lymphoblasts and multiple myeloma cells (Lameris et al., 2016).

CD1d has also been shown to control inflammatory responses in myeloid cells through cell-intrinsic mechanisms independent of NKT cells or CD1d ligation, although results in this area have been controversial (Fig. 3). Initial work suggested that CD1d functions as a positive regulator of inflammatory responses, as CD1d-deficient macrophages displayed decreased secretion of inflammatory cytokines after Toll-like receptor (TLR) stimulation (Liu et al., 2019) (Fig. 3A). This effect was attributed to binding of the natural ligand glycosphingolipid iGb3 to CD1d and CD1d reverse signalling dependent on Tyr^332^ phosphorylation of the CD1d intracellular tail. These results have been later disputed (Cui et al., 2020), as a recent report suggests that CD1d-intrinsic signalling negatively regulates inflammatory responses in macrophages. In this context, the authors propose that iGb3 binding to CD1d triggers dephosphorylation of the Ser^330^ in the CD1d intracellular tail resulting in transcriptional downregulation of the *Nlrp3* inflammasome (Cui et al., 2020) (Fig. 3B). The factors underlying these seemingly contradictory results remain unclear, although the different CD1d-KO mouse lines (C.129S2-Cd1tm1Gru/J *vs.* Del(3Cd1d2-Cd1d1)1Sbp), different experimental cells (thioglycolate induced macrophages *vs.* bone-marrow derived macrophages) and even environmental factors (microbiota, diet) could contribute to this discrepancy. To settle this controversy, recent work from our lab demonstrates -using two independently generated CD1d-KO strains (kept in different animal facilities): Del(3Cd1d2-Cd1d1)1Sbp and CD1d^flox^xPGK^Cre^ (Gaya et al., 2018, Saez de Guinoa et al., 2018)- that CD1d functions as a negative regulator of TLR responses (Brailey et al., 2022). In fact, CD1d-KO primary peritoneal macrophages and bone-marrow derived dendritic cells from both these mouse lines secrete more type I interferon and pro-inflammatory cytokines than WT cells when stimulated with a variety of TLR ligands (Fig. 3C). This regulatory function of CD1d is cell intrinsic (as it is detected in CD1d-deficient macrophages isolated from mixed bone-marrow chimeras) and it is related to metabolic perturbations as discussed below.

## 5 Cell-intrinsic functions of CD1d: metabolic regulation

While the capacity of CD1d to bind lipid antigens has been described decades ago, only recently data has emerged supporting the notion that CD1d can control lipid metabolic programs in CD1d-expressing cells, functioning as a molecular link between lipid metabolism and immunity (Brailey et al., 2022). In addition to its lipid-binding capacity, the CD1d molecule *per se* has certain characteristics that suggest a possible link with lipid metabolic programs. For instance, molecules involved in CD1d trafficking and lipid-loading, are also directly involved in lipid metabolism. As such, microsomal triglyceride transfer protein which loads lipids into CD1d in the ER, also transfers triglycerides, cholesterol and phospholipids to nascent apolipoprotein B (Hooper et al., 2015). Moreover, while CD1d is constitutively expressed in many cell types, its expression can be regulated by transcription factors such as peroxisome proliferator-activated receptor gamma (PPARγ)(Szatmari et al., 2006) and delta (PPARδ)(Adhikary et al., 2015), which are lipid-activated regulators of metabolic programs. Recent work from our lab provides evidence for a direct role for CD1d as a cell-intrinsic regulator of lipid metabolism in macrophages (Fig. 3C). Accordingly, we found that CD1d-deficient macrophages exhibit a metabolic reprograming with a transcriptional downregulation of lipid synthesis pathways and an increase in lipid uptake, associated with the reduced expression of the transcription factor PPARδ. This CD1d-dependent metabolic regulation is cell intrinsic and independent of CD1d ligation or NKT cells. Mechanistically, CD1d modulates lipid import by controlling lipid uptake through the transporter CD36. Interestingly, these metabolic alterations underpin the increased proinflammatory responses of CD1d-KO macrophages, as blocking lipid uptake through CD36 restores metabolic and immune responses. Moreover, in this context, blocking surface CD1d in WT cells (with a monoclonal antibody, 19G11), is sufficient to increase CD36-mediated lipid uptake as well as proinflammatory responses after TLR stimulation. Thus, these results support a model by which the metabolic rewiring controlled by CD1d, primes cells for hyper-responsiveness to TLR ligands (Brailey et al., 2022). Whist the mechanisms by which CD1d modulates CD36-mediated lipid uptake are not yet defined, both molecules are located in lipid rafts at the plasma membrane (Eyre et al., 2007, Park et al., 2005), and they could cooperate to regulate lipid uptake and/or lipid delivery to intracellular compartments. Interestingly, a recent report identified CD36 as a ligand for human CD1d tetramers (Gherardin et al., 2021), further supporting the idea of a CD1d-CD36 axis as a key factor in the regulation of lipid metabolic programs. Moreover, and non-mutually exclusive, CD1d could also compete with CD36 for lipid binding either at the cell surface or in intracellular compartments, consequently altering lipid availability and regulating cellular lipid metabolism. In support of this, it has been suggested that CD1d could bind cholesterol-derivatives in the cell surface ultimately leading to PPARδ activation (Rosales et al., 2015). Thus, since lipid metabolic programs are critical in controlling immune cell function and activation, the capacity of CD1d to shape metabolic programs in macrophages opens new pathways to investigate unappreciated functions for this molecule in metabolic, inflammatory or infectious diseases.

## 6 Unconventional functions of CD1d: implication for disease and opportunities for therapy

A vast amount of literature supports a central role for CD1d/NKT cells in a variety of disease contexts, including inflammatory, infectious or autoimmune diseases (Mori et al., 2016). However, the biological and functional importance of different NKT cell subsets (type I *vs.* type II) in disease has often been deduced by comparing Jα18-KO mice (which lack type I NKT cells only) with CD1d-KO mice (which lack both type I and II NKT cells, as well as CD1d expression). In scenarios where phenotypes are observed in CD1d-KO mice but not in Jα18-KO mice, those have been subsequently attributed to the function of type II NKT cells. However, in light of the APC-intrinsic CD1d functions described above, it is difficult to differentiate the contribution of type II NKT cells *vs.* intrinsic CD1d and consequently, NKT-dependent conclusions made in these studies may need to be re-evaluated. Thus, further studies are granted to determine the regulation of cellular functions by CD1d and its implications in the control of tissue homeostasis and disease.

One of the key pathologies where intrinsic CD1d functions have been proposed to play a critical role is in IBD, although data suggest complex functions for this molecule likely related to cell-specific and disease-specific effects. Largely, IBD can be classified in two major forms of disease -ulcerative colitis (UC) and Crohn’s disease (CD)- which differ in the extent of pathology and in their immune profile (Xavier and Podolsky, 2007). In general, CD1d/NKT cells have been suggested to play distinct and opposing roles in UC *vs.* CD (Brailey et al., 2020). Intestinal NKT cells contribute to the fuelling of intestinal inflammation in UC possibly through secretion of IL-13. Accordingly, in the oxazolone model of murine UC, there is striking protection from disease in CD1d-deficient mice or WT mice pre-treated with anti-CD1d or anti-IL-13 blocking antibodies (Heller et al., 2002, Olszak et al., 2012). This effect is however dependent on the cell-specific expression of CD1d. While expression of CD1d in hematopoietic cells seems to mediate NKT cell activation and to fuel inflammation, intrinsic CD1d signalling in IECs drives IL-10 secretion and protection from disease (Olszak et al., 2014). On the other hand, the literature surrounding the role of CD1d/NKT in the pathogenesis of CD -using the murine model of dextran sodium sulfate-induced (DSS)-mediated colitis- is controversial, with studies showing either protective or detrimental effects (Lee et al., 2018, Saubermann et al., 2000). The factors underlying these inconsistencies remain unclear, but alterations in the microbiota of CD1d-KO strains have been proposed to have a critical influence in the outcome of DSS colitis (Selvanantham et al., 2016). Moreover, it is likely that the cell-specific functions of CD1d also influence disease outcome. Accordingly, it has been shown that macrophage-specific CD1d outside-in signalling is detrimental for DSS-induced colitis progression, as CD1d intracellular signalling inhibits NLRP3 inflammasome, which in turn regulates gut-blood barrier integrity and intestinal homeostasis (Cui et al., 2020). Moreover, it is worth noting that CD1d crosslinking in ILC3s induces secretion of IL-22 (Saez de Guinoa et al., 2017) which is known to support epithelial barrier function and has been shown to be protective in DSS-induced colitis (Keir et al., 2020). Together, these data suggest distinct and cell-specific roles for CD1d in the development of IBD and highlight the importance of assessing the CD1d-dependent signalling pathways contributing to disease development and progression.

The capacity of CD1d to shape lipid metabolic programs in macrophages (Brailey et al., 2022) sheds new light onto unappreciated functions for CD1d which could regulate the outcome of a variety of immune responses. Indeed, an immune-metabolic axis has been found to be a fundamental driver for immunity in many diseases (Jung et al., 2019). For instance, many reports highlight an intimate link between lipid metabolism and host immunity to viral and bacterial infections (Araldi et al., 2017, Blanc et al., 2013, Blanc et al., 2011, Teng et al., 2017, York et al., 2015), as well as during the induction of trained immunity in myeloid cells (Bekkering et al., 2018, Mitroulis et al., 2018). Also, lipid metabolic pathways are closely linked to macrophage polarization (Wculek et al., 2022) and this in turn can be modulated by PPARδ (Kang et al., 2008, Odegaard et al., 2008). The role of CD1d/NKT cells has been well established in many of these immune processes, including viral and bacterial infections, tumour progression or macrophage polarization in obesity or cancer (Cruz et al., 2022, Vogt and Mattner, 2021). However, the contribution of CD1d-dependent metabolic regulation to disease outcome or progression has never been considered and requires further investigation.

Given the capacity of CD1d to modulate lipid metabolism and inflammatory responses (Brailey et al., 2022), it is likely that its cell-intrinsic functions could play a role in the progression of metabolic diseases including obesity. A plethora of studies have been conducted which associate the CD1d-NKT signalling axis with diet-induced obesity resulting in either disease protection or progression described for CD1d-deficient *vs.* WT mice (Satoh and Iwabuchi, 2018). While differences in mouse strains and microbiota, as well as diet composition may partially underpin the conflicting results, the contribution of intrinsic CD1d signalling in adipose tissue inflammation cannot be ruled out. Accordingly, it is important to point out that some of the metabolic alterations described in CD1d-KO mice are not recapitulated (or found to be milder) in Jα18-KO mice (lacking iNKT cells), suggesting a role for CD1d in metabolic regulation which may happen independently of iNKT cells (Kotas et al., 2011). Also, in this context it is essential to consider the cell-specific functions of CD1d. Both adipocytes and adipose macrophages express CD1d and they have been reported to interact with NKT cells in adipose tissue and promote diet-induced obesity-associated inflammation (Huh et al., 2017, Satoh et al., 2016, Satoh et al., 2022, Zhang et al., 2018). Unveiling the specific CD1d-intrinsic *vs.* NKT-dependent functions of individual CD1d-expressing cell types in adipose tissue may help unpick the conflicting literature surrounding the CD1d-NKT axis in metabolic disease.

Mutations in the intracellular tail of CD1d molecules have been associated with altered intracellular recirculation of CD1d, accumulation of CD1d on the cell surface and defective iNKT development and activation (Chiu et al., 2002, Shin and Park, 2014). However, it is important to consider that such mutations may have wider implications in homeostasis and disease by altering the intrinsic signalling capacity of CD1d. The CD1d cytoplasmic tail is crucial for CD1d-retrograde signalling at least in epithelial cells (and possibly in other CD1-expressing cells). Accordingly, while crosslinking of CD1d induces STAT3 phosphorylation and IL-10 secretion in IECs, impaired STAT3 phosphorylation was observed in IECs from mice deficient in the CD1d-tail, suggesting that this region is critical for the protective effect of CD1d-retrograde signalling in intestinal inflammation (Olszak et al., 2014). Another crucial element supporting a function for CD1d in disease is the fact that the levels of expression of CD1d have been shown to be altered in a number of autoimmune diseases, such as systemic lupus erythematosus (Bosma et al., 2012), psoriasis (Bonish et al., 2000), rheumatoid arthritis (Kojo et al., 2003) or IBD (Perera et al., 2007), as well as in viral (Moll et al., 2010) or bacterial (Raghuraman et al., 2006) infections and during tumour progression (Nair and Dhodapkar, 2017). Interestingly, the CD1d tail is critical for CD1d downregulation at least in response to several infections such as HIV (Chen et al., 2006) or *Chlamydia trachomatis* (Kawana et al., 2007). In these contexts, it is likely that changes in CD1d expression levels will regulate the activation of NKT cells. However, it is also possible that the down (or up) regulation of CD1d itself contributes to modulate the activation, function or survival of CD1d-expressing cells, and consequently will shape the development of the immune response. In these scenarios, the use of antibodies or other approaches targeting CD1d expression, trafficking or signalling could provide novel therapeutic strategies for a variety of inflammatory, autoimmune and infectious diseases. Indeed, reports have shown the capacity of αCD1d antibodies to modulate the outcome of immune responses *in vivo*. For instance, injection of αCD1d antibodies in mice enhanced systemic IL-12, IFN-γ and IFN-α, and was protective against infection by encephalomyocarditis virus (Yue et al., 2010). Another interesting possibility would be to incorporate αCD1d antibodies in tumour immunotherapy. Tumour cells expressing CD1d are mainly of myelomonocytic and B cell origin, while very few solid tumours express CD1d (Nair and Dhodapkar, 2017). However, cytokine secretion induced in CD1d+ APCs following injection of αCD1d antibodies, has been shown to be sufficient to inhibit the growth of different CD1d-negative experimental carcinomas in mice (Teng et al., 2009). Moreover, *in vitro* experiments showed that CD1d ligation can cooperate with other anti-myeloma agents to induce cell death (Spanoudakis et al., 2009), raising the possibility of incorporating αCD1d antibody treatment to the arsenal of therapies targeting early-stage myeloma. Thus, collectively, these studies suggest that manipulating CD1d-dependent signalling could alter the activation/survival of CD1d-expressing cells *in vivo* providing potential therapeutic options to manipulate immune responses and regulate the progression of a variety of diseases.

## 7 Group 1 CD1 molecules: unconventional functions?

As the only CD1 molecule expressed in murine cells, the “unconventional” functions have been mainly studied for CD1d, but it is possible that are conserved among other CD1 isoforms. Human cells express CD1e (an intracellular lipid transfer protein), as well as membrane-bound CD1a, CD1b, CD1c and CD1d. Indeed, in human immune cells group 1 membrane-bound CD1 isoforms (CD1a-c) are expressed at greater levels than and/or in conjunction with CD1d and are highly inducible by cytokines and microbial products. As with CD1d, CD1a-c molecules are folded in the ER, and bind β2-microblobulin and self-lipids prior to transiting through the secretory pathway to the cell surface. Tyrosine-based (YXXZ) cytoplasmic tail motifs are also present in CD1b (two motifs) and CD1c (one motif), but not in CD1a dictating differences in the endosomal-recycling pathways for these molecules. As such, CD1a is the only CD1 molecule that does not traffic to the endolysosome (Vartabedian et al., 2016).

There is some evidence that suggest the involvement of group 1 CD1 isoforms in “unconventional” functions, both related to CD1 retrograde signalling as well as metabolic regulation. On the one hand, CD1d-retrograde signalling on epithelial cells is dependent on the cytoplasmic tail (Colgan et al., 1999, Olszak et al., 2014). While crosslinking of CD1d is sufficient to induce IL-10 secretion by IECs, crosslinking of a chimeric molecule expressing extracellular CD1d and cytoplasmic CD1a did not induce IL-10 (Colgan et al., 1999). Given that cytoplasmic tail motifs (YXXZ) are also present in CD1b and CD1c it is possible that a similar mechanism for retrograde signalling operates in these molecules. On the other hand, data would also support a role for CD1b and CD1c molecules in the regulation of metabolic pathways in APCs. Some of the available evidence for possible unconventional functions of group 1 CD1 molecules, includes the fact that CD36 is a ligand for human CD1b, CD1c and CD1d tetramers (but not for CD1a tetramers) (Gherardin et al., 2021). This could suggest that CD1b and CD1c could also potentially modulate CD36 functions and metabolic programs in human APCs, particularly on cells in which their expression may be higher than CD1d. On the other hand, CD1a tetramers do not bind CD36 and CD1a is the only molecule which doesn’t travel through the lysosomes. It is important to note that CD1d-dependent regulation of CD36 is independent of the CD1d cytoplasmic tail (Brailey et al., 2022), but metabolic regulation may still require recirculation through specific intracellular compartments. This would be consistent with a model in which CD1b-d molecules may interact or compete with CD36 for lipid binding in intracellular compartments, consequently altering lipid availability and regulating cellular lipid metabolism.

## 8 Conclusions

While the antigen-presenting function of CD1d is well established, it is becoming clear that CD1d can have additional “unconventional” functions which play a key role in controlling the activation of CD1d-expressing cells, ultimately modulating cytokine secretion, survival or metabolic programs in immune and non-immune cells. Such roles can in some cases be mediated by CD1d-retrograde signalling, yet in other circumstances they are cell intrinsic and lead to metabolic regulation of APCs. While the relevance of these non-classical CD1d functions has been mainly investigated *in vitro*, some studies also support key roles for CD1d in controlling inflammation, infection or tumour progression *in vivo*. Moreover, the fact that CD1d expression levels are altered in a variety of human disorders suggest additional links between CD1d functions and disease pathophysiology. While it is likely that these changes in CD1d levels can control NKT cell activation, they may also modulate cellular metabolism and activation ultimately shaping disease progression. Moreover, while data about the “unconventional” functions of group 1 CD1 molecules is scarce, it is likely that CD1b and CD1c molecules also contribute to both retrograde signalling and metabolic regulation, particularly in cell types in which expression of these molecules is higher than that of CD1d. Thus, manipulating CD1 functions could become a novel therapeutic strategy for treatment of a variety of autoimmune, metabolic or infectious diseases.

## CRediT authorship contribution statement

**Lauren Evans:** Conceptualization, Writing – original draft, Writing – review & editing. **Patricia Barral:** Conceptualization, Funding acquisition, Supervision, Writing – original draft, Writing – review & editing.

## Data Availability

No data was used for the research described in the article.
